# Supplementary material for: Functional Conservation and Divergence of Five AP1/FUL-like Genes in Marigold (Tagetes erecta L.)
Source: Genes (Basel). 2021 Dec 17;12(12):2011. doi: 10.3390/genes12122011 (PMC8700864; doi:10.3390/genes12122011)
Supplement: Supplementary file 1 [file genes-12-02011-s001.zip › genes-1470922-supplementary.pdf]

Table S1. Sequences of primers.

Primer	Sequence (5'-3')	Remarks
TeAP1-1-full-F	TCTCACCTGCCACCAAACCTA	Full-length gene cloning
TeAP1-1-full-R	TCACCGGCTTGTCGATCAGT	Full-length gene cloning
TeAP1-2-full-F	ACTCAACCCACCTCACCTACACA	Full-length gene cloning
TeAP1-2-full-R	GGCTCACTAGATCATTTTACGAAGG	Full-length gene cloning
TeFUL1-full-F	CGGAGATAGAAATGGGACGG	Full-length gene cloning
TeFUL1-full-R	ATACTACGGTGTCCAAACGT	Full-length gene cloning
TeFUL2-full-F	TAAGATGGGAAGAGGCAGAGTG	Full-length gene cloning
TeFUL2-full-R	GGCCTTCGGATTTTACTGGTTA	Full-length gene cloning
TeFUL3-full-F	TAAGCGGTGGTTGTTTCCGG	Full-length gene cloning
TeFUL3-full-R	CCGGCCGATAAAATTACATGCACAT	Full-length gene cloning
RT-*ACT*-F	GGGAAATGAATGCCAAAGCCAAG	QRT-PCR，Semi-PCR
RT-*ACT*-R	AAGACTTCACAACCACTCTCCAACT	QRT-PCR，Semi-PCR
qRT-*TeAP1-1*-F	GACACCGATACAACCTTCCTCA	QRT-PCR，Semi-PCR
qRT-*TeAP1-1*-R	CATCACCTCCTTCGCCGCTA	QRT-PCR，Semi-PCR
qRT-*TeAP1-2*-F	ATGCTGGCGGTGGTGGTGAA	QRT-PCR，Semi-PCR
qRT-*TeAP1-2*-R	TCATTTTACGAAGGAAAGCACC	QRT-PCR，Semi-PCR
qRT-*TeFUL1*-F	AGGAGAAAGAGTTAGAACAACAGGC	QRT-PCR，Semi-PCR
qRT-*TeFUL1*-R	GGTTGACCTTCTCTTGGGTTCTC	QRT-PCR，Semi-PCR
qRT-*TeFUL2*-F	CCAGCAGTGGACCATTCATTG	QRT-PCR
qRT-*TeFUL2*-R	GCGGCAGATACATTATGAGTTTGAG	QRT-PCR
qRT-*TeFUL3*-F	CTTATGGACCAGCACTCACAT	QRT-PCR，Semi-PCR
qRT-*TeFUL3*-R	GCATTTCTTCTGTTTCTCCGTTAC	QRT-PCR，Semi-PCR
RT-*TeFUL2*-F	ATAAGGCACTATGCTGGGGAAGA	Semi-PCR
RT-*TeFUL2*-R	GCGGATCATCCATAGCGGAA	Semi-PCR
AD-F	CTATTCGATGATGAAGATACCCCACCAAACCC	Yeast two-hybrid
AD-R	GTGAACTTGCGGGGTTTTTCAGTATCTACGATT	Yeast two-hybrid
BD-F	TCATCGGAAGAGAGTAG	Yeast two-hybrid
BD-R	GAGTCACTTTAAAATTTGTAT	Yeast two-hybrid
AD/BD-*TeAP1-1* -F	**CATATG**ATGGGTAGAGGTAAGGTACA	Yeast two-hybrid
AD/BD-*TeAP1-1* -R	**GAATTC**TTATGAAGGAAAACACCTCA	Yeast two-hybrid
AD/BD-*TeAP1-2* -F	**CATATG**ATGGGTAGAGGTAAGGTACA	Yeast two-hybrid
AD/BD-*TAP1-2* -R	**GAATTC**TTACGAAGGAAAGCACCTCA	Yeast two-hybrid
AD/BD-*TeFUL1* -F	**CATATG**ATGGGAAGAGGAAGAGTAACATTGA	Yeast two-hybrid
AD/BD-*TeFUL1*-R	**GAATTC**TTATATAATATGATCTACTGGCCGG	Yeast two-hybrid
AD/BD-*TeFUL2* -F	**CATATG**ATGGGAAGAGGCAGAGTGC	Yeast two-hybrid
AD/BD-*TeFUL2* -R	**GAATTC**TTACTGGTTAATGTGGCGGA	Yeast two-hybrid
AD/BD-*TeFUL3* -F	**CATATG**ATGGGAAGAGGAAGAGTAACATTGA	Yeast two-hybrid
AD/BD-*TeFUL3* -R	**GAATTC**TTATATAATATGATCTACTGGCCGG	Yeast two-hybrid
AD/BD-*TeSEP1*-F	**CATATG**ATGGGTAGAGGAAGAGTTGAATTGA	Yeast two-hybrid
AD/BD-*TeSEP1*-R	**GGATCC**TCAAAGCATCCATCCAGGGA	Yeast two-hybrid
AD/BD-*TeSEP3-1*-F	**CATATG**ATGGGAAAAGGAAGGTTAGAGTTGA	Yeast two-hybrid
AD/BD-*TeSEP3-1*-R	**GGATCC**TCATGCTGGCCAACCCTG	Yeast two-hybrid

AD/BD-*TeSEP3-2*-F	**CATATG**ATGGGAAGAGGAAGAGTAGAG	Yeast two-hybrid
AD/BD-*TeSEP3-2*-R	**GAATTC**TTAACATGGCATCCATCCTT	Yeast two-hybrid
AD/BD-*TeSEP3-3*-F	**CATATG**ATGGGGAGAGGAAGA	Yeast two-hybrid
AD/BD-*TeSEP3-3*-R	**GAATT**CTCACTGAAACCATATTGACATGTAA	Yeast two-hybrid
AD/BD-*TeSEP4*-F	**CATATG**ATGGGAAGAGGCAGAGTTGAAC	Yeast two-hybrid
AD/BD-*TeSEP4*-R	**GGATCC**CTTATTTCTTAAGGCTTTATTTGTT	Yeast two-hybrid
AD/BD-*TeAGL6*-F	**CATATG**ATGGGGAGAGGACGAGTCG	Yeast two-hybrid
AD/BD-*TeAGL6*-R	**GGATCC**TCAAAGATTAACCCATCCATGGATA	Yeast two-hybrid
35S-TeAP1-1-F	**GGTACC**ATGGGTAGAGGTAAGGTACA	Ectopic expression vector
35S-TeAP1-1-R	**GGATCC**TTATGAAGGAAAACACCTCA	Ectopic expression vector
35S-TeAP1-2-F	**GGTACC**ATGGGTAGAGGTAAGGTACA	Ectopic expression vector
35S-TeAP1-2-R	**GTCGAC**TTACGAAGGAAAGCACCTCA	Ectopic expression vector
35S-TeFUL1-F	**CCCGGG**ATGGGACGGGGAAAGGTAAC	Ectopic expression vector
35S-TeFUL1-R	**TCTAGA**TTACTTGCTAATGTGTTGAAG	Ectopic expression vector
35S-TeFUL2-F	**GGTACC**ATGGGAAGAGGCAGAGTGC	Ectopic expression vector
35S-*TeFUL2*-R	**GTCGAC**TTACTGGTTAATGTGGCGGA	Ectopic expression vector
35S-TeFUL3-F	**GGTACC**ATGGGAAGAGGAAGAGTAACATTGA	Ectopic expression vector
35S-TeFUL3-R	**GTCGAC**TTATATAATATGATCTACTGGCCGG	Ectopic expression vector
qRT-*FT*-F	TATCCCTGCTACAACTGGAACAACC	QRT-PCR
qRT-*FT*-R	GCCTGCCAAGCTGTCGAAACAATAT	QRT-PCR
qRT-*LFY*-F	TACTCTCCGCCGCTGGTGATTC	QRT-PCR
qRT-*LFY*-R	ACTTCCTCCTCCGCCGTTATTCC	QRT-PCR
qRT-*SCO1*-F	TGAAAGCGAAGTTTGGTCAAATAAGA	QRT-PCR
qRT-*SCO1*-R	TCTTGAAGAACAAGGTAACCCAATGA	QRT-PCR
qRT-*SEP3*-F	GTATCAGGGGCAACAAGATGGAAT	QRT-PCR
qRT-*SEP3*-R	AAAGAGAGGGATTGATTAAGTGAGAAAGA	QRT-PCR
qRT-*AP1*-F	AAATCCAGCATCCTTACATGCTCTC	QRT-PCR
qRT-*AP1*-R	CAGTTCGAGATCATTCCTCCTCATT	QRT-PCR
qRT-*SPL9*-F	CAAGGTTCAGTTGGTGGAGGA	QRT-PCR
qRT-*SPL9*-R	TGAAGAAGCTCGCCATGTATTG	QRT-PCR
qRT-*SVP*-F	GAAGGACAGTCGTCGGAGTC	QRT-PCR
qRT-*SVP*-R	GCCTCTTCCATAGGCAGAAA	QRT-PCR
qRT-*TFL1*-F	GCACAACAGATGCTACGTTTGGC	QRT-PCR
qRT-*TFL1*-R	CCTATGCTTGGCCTTGGCAATTC	QRT-PCR
qRT-*AGL24*-F	GAAGGCTTTGGAGACAGAGTCGGTGA	QRT-PCR
qRT-*AGL24*-R	AGATGGAAGCCCAAGCTTCAGGGAA	QRT-PCR

Note: bold font is the sequence of enzyme cutting site. *TeAG1* (Acc. No. MT452648) and *TeAG2* (Acc. No. MT452649) are class C genes; *TeAGL11-1* (Acc. No. MT394168) and *TeAGL11-2* (Acc. No. MT394169) are class D genes; *TeSEP1* (Acc. No. MT394175), *TeSEP3-1* (Acc. No. MT394176), *TeSEP3-2* (Acc. No. MT394177), *TeSEP3-3* (Acc. No. MT394178), *TeSEP4* (Acc. No. MT394179) are class E genes. *TeAGL6* (Acc. No. MT394180) is *AGL6*-like gene.

Table S2. Sequence information of AP1/FUL-like proteins used to construct evolutionary tree.

No.	Protein Name	Accession No.	Species	Plant Lineage
1	TeAP1-1	MT394170	*Tagetes erecta*	Asteraceae
2	TeAP1-2	MT394171	*Tagetes erecta*	Asteraceae
3	TeFUL1	MT394172	*Tagetes erecta*	Asteraceae
4	TeFUL2	MT394173	*Tagetes erecta*	Asteraceae
5	TeFUL3	MT394174	*Tagetes erecta*	Asteraceae
6	HaFUL	XP_021983861	*Helianthus annuus*	Asteraceae
7	HAM75	AF462152	*Helianthus annuus*	Asteraceae
8	HAM92	AY173071	*Helianthus annuus*	Asteraceae
9	CDM8	AAO22981	*Chrysanthemum morifolium*	Asteraceae
10	CDM41	AAO22980	*Chrysanthemum morifolium*	Asteraceae
11	CDM111	AAO22979	*Chrysanthemum morifolium*	Asteraceae
12	GSQUA1	CAA08805	*Gerbera hybrida*	Asteraceae
13	GSQUA2	CAX65661	*Gerbera hybrida*	Asteraceae
14	GSQUA3	CAX65662	*Gerbera hybrida*	Asteraceae
15	GSUQA5	CAX65663	*Gerbera hybrida*	Asteraceae
16	GSQUA6	CAX65664	*Gerbera hybrida*	Asteraceae
17	EuFUL	XP_022016988	*Antirrhinum majus*	Scrophulariaceae
18	DEFH28	AY040247	*Antirrhinum majus*	Scrophulariaceae
19	SQUA	X63701	*Antirrhinum majus*	Scrophulariaceae
20	AP1	NP_177074	*Arabidopsis thaliana*	Brassicaceae
21	CAL	NM_102395	*Arabidopsis thaliana*	Brassicaceae
22	FUL	NP_568929	*Arabidopsis thaliana*	Brassicaceae
23	PFG	AF176782	*Petunia hybrida*	Solanaceae
24	FBP26	AF176783	*Petunia hybrida*	Solanaceae
25	FBP29	AF335245	*Petunia hybrida*	Solanaceae
26	PhFUL	AY306172	*Petunia hybrida*	Solanaceae
27	CanMADS6	AF130118	*capsicum annuum*	Solanaceae
28	DcMADS1	AJ271147	*Daucus carota*	Apiaceae
29	OsMADS14	AF058697	*Oryza sativa*	Poaceae
30	OsMADS15	AF058698	*Oryza sativa*	Poaceae
32	ZAP1	L46400	*Zea mays*	Poaceae
33	TrmAP1	AY188331	*Triticum monococcum*	Poaceae
34	ORAP1-2	DQ104327	*Phalaenopsis amabilis*	Orchidaceae
35	DthyrFL1	AY927236	*Dendrobium thyrsiflorum*	Orchidaceae
36	AlFL	AY306138	*Allium sp.*	Liliaceae
37	GRCD2	CAH04878.1	*Gerbera hybrida*	Asteraceae
38	GRCD4	CAX65570.1	*Gerbera hybrida*	Asteraceae
39	PhAGL6	BAA94287.1	*Petunia hybrida*	Solanaceae
40	AGL6	NP_182089.1	*Arabidopsis thaliana*	Brassicaceae

Table S3. Amino acid sequence alignment of TeAP1/FUL class proteins.

	TeAP1-1	TeAP1-2	TeFUL1	TeFUL2	TeFUL3
TeAP1-1	100%	89.92%	51.81%	49.80%	51.00%
TeAP1-2	89.92%	100%	49.19%	52.02%	49.60%
AP1	59.39%	59.14%	48.05%	53.52%	46.09%
CAL	55.00%	54.86%	42.35%	51.76%	43.53%
TeFUL1	51.81%	49.19%	100%	54.04%	61.16%
TeFUL2	49.80%	52.02%	54.04%	100%	50.21%
TeFUL3	51.00%	49.60%	61.16%	50.21	100%
FUL	54.25%	54.44%	52.89%	54.55%	54.07%
AGL79	44.84%	45.24	46.18%	49.40%	42.58%

Note: the proteins AP1, FUL, CAL, and AGL79 are from Arabidopsis. TeAP1-1, TeAP1-2, TeFUL1, TeFUL2, and TeFUL3 were from marigold.

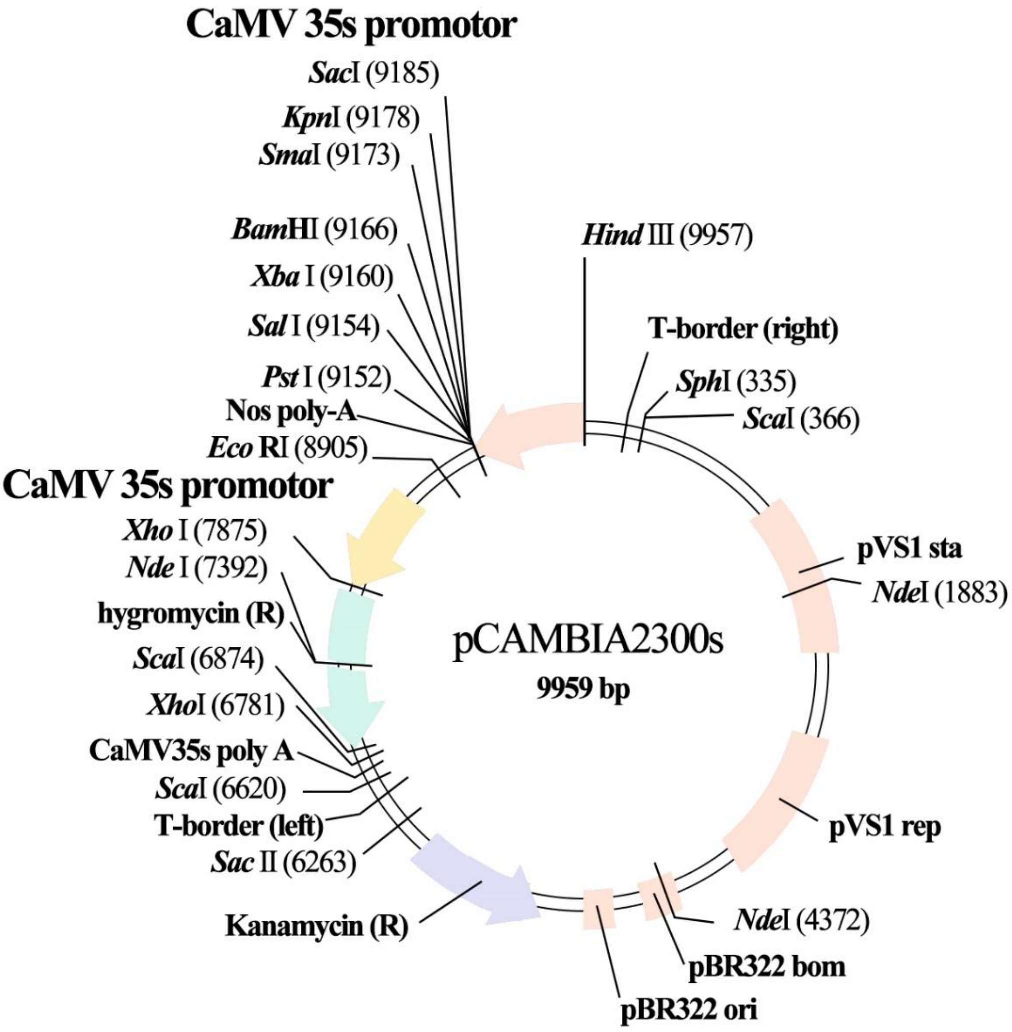

Figure S1. The map of pCAMBIA2300s.

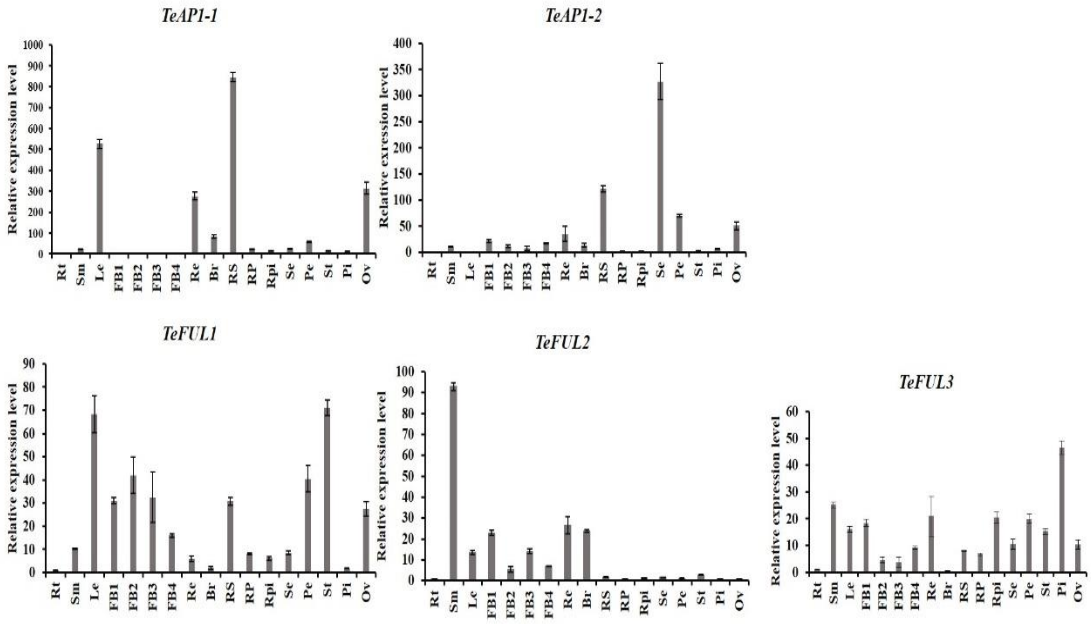

Figure S2. Expression levels of *TeAP1/FUL* in different tissues and organs. Rt: root; Sm: stems; Le: leaf; FB1-FB4: flower buds were 0-1mm, 2-3mm, 4-5 mm and 6-7mm in diameter, respectively; Re: receptacle; Br: bract; RS: sepal of ray floret; RP: petal of ray floret; RPi: pistil of ray floret; Se: sepal of disk floret; Pe: petal of disk floret; St: stamen of disk floret; Pi: pistil of disk floret; Ov: ovary.

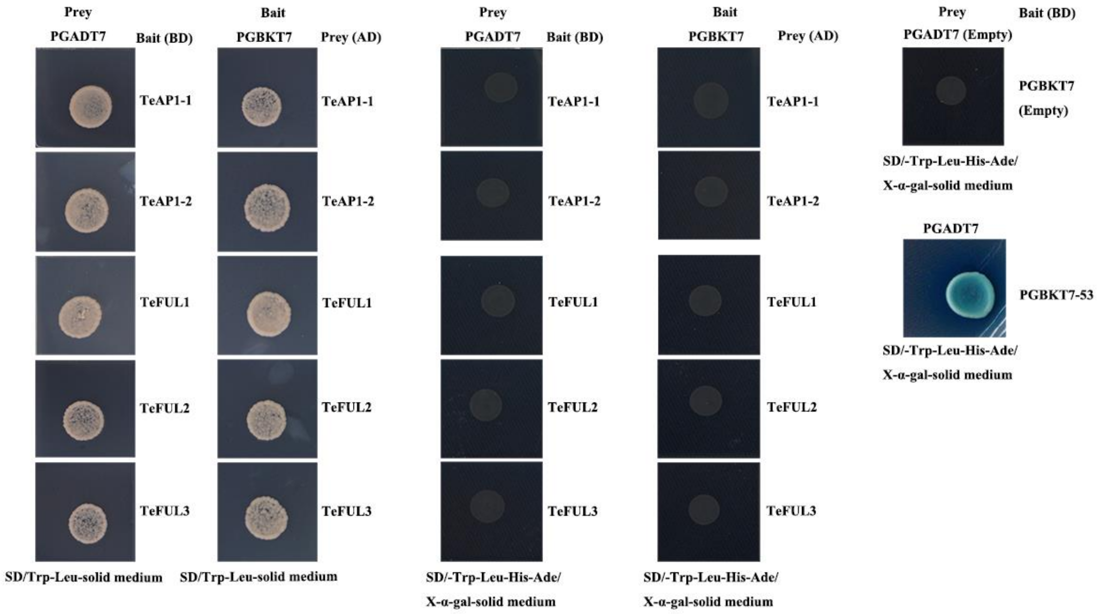

(a)

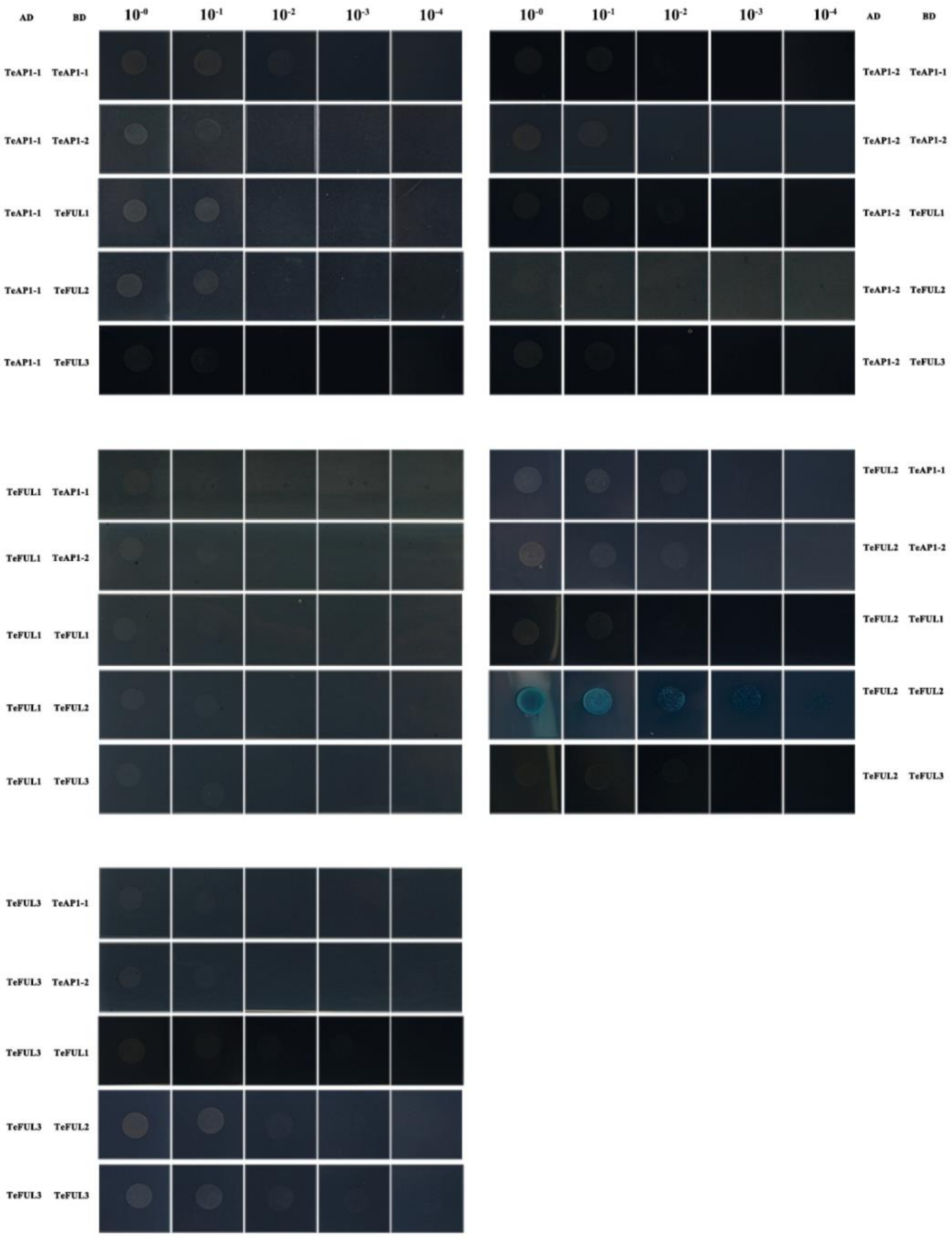

AD
BD
10-0
10-1
10-2
10-3
10-4
TeAP1-1 TeAP1-1
TeAP1-1 TeAP1-2
TeAP1-1 TeFUL1
TeAP1-1 TeFUL2
TeAP1-1 TeFUL3
TeAP1-2 TeAP1-1
TeAP1-2 TeAP1-2
TeAP1-2 TeFUL1
TeAP1-2 TeFUL2
TeAP1-2 TeFUL3
TeFUL1 TeAP1-1
TeFUL1 TeAP1-2
TeFUL1 TeFUL1
TeFUL1 TeFUL2
TeFUL1 TeFUL3
TeFUL2 TeAP1-1
TeFUL2 TeAP1-2
TeFUL2 TeFUL1
TeFUL2 TeFUL2
TeFUL2 TeFUL3
TeFUL3 TeAP1-1
TeFUL3 TeAP1-2
TeFUL3 TeFUL1
TeFUL3 TeFUL2
TeFUL3 TeFUL3

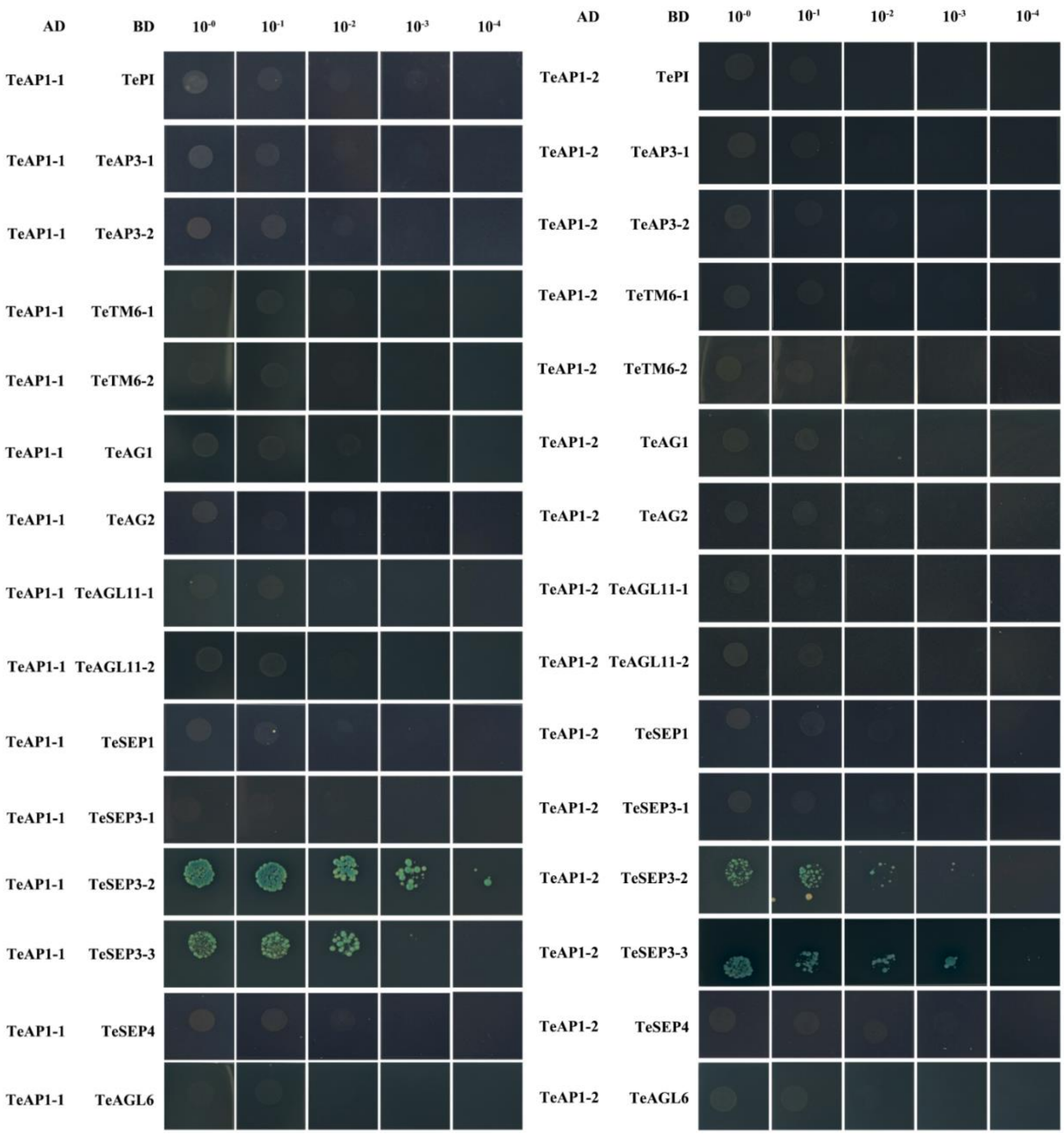
AD
BD
10^{-0}
10^{-1}
10^{-2}
10^{-3}
10^{-4}
TeAP1-1 TePI
TeAP1-1 TeAP3-1
TeAP1-1 TeAP3-2
TeAP1-1 TeTM6-1
TeAP1-1 TeTM6-2
TeAP1-1 TeAG1
TeAP1-1 TeAG2
TeAP1-1 TeAGL11-1
TeAP1-1 TeAGL11-2
TeAP1-1 TeSEP1
TeAP1-1 TeSEP3-1
TeAP1-1 TeSEP3-2
TeAP1-1 TeSEP3-3
TeAP1-1 TeSEP4
TeAP1-1 TeAGL6
AD
BD
10^{-0}
10^{-1}
10^{-2}
10^{-3}
10^{-4}
TeAP1-2 TePI
TeAP1-2 TeAP3-1
TeAP1-2 TeAP3-2
TeAP1-2 TeTM6-1
TeAP1-2 TeTM6-2
TeAP1-2 TeAG1
TeAP1-2 TeAG2
TeAP1-2 TeAGL11-1
TeAP1-2 TeAGL11-2
TeAP1-2 TeSEP1
TeAP1-2 TeSEP3-1
TeAP1-2 TeSEP3-2
TeAP1-2 TeSEP3-3
TeAP1-2 TeSEP4
TeAP1-2 TeAGL6

(b)

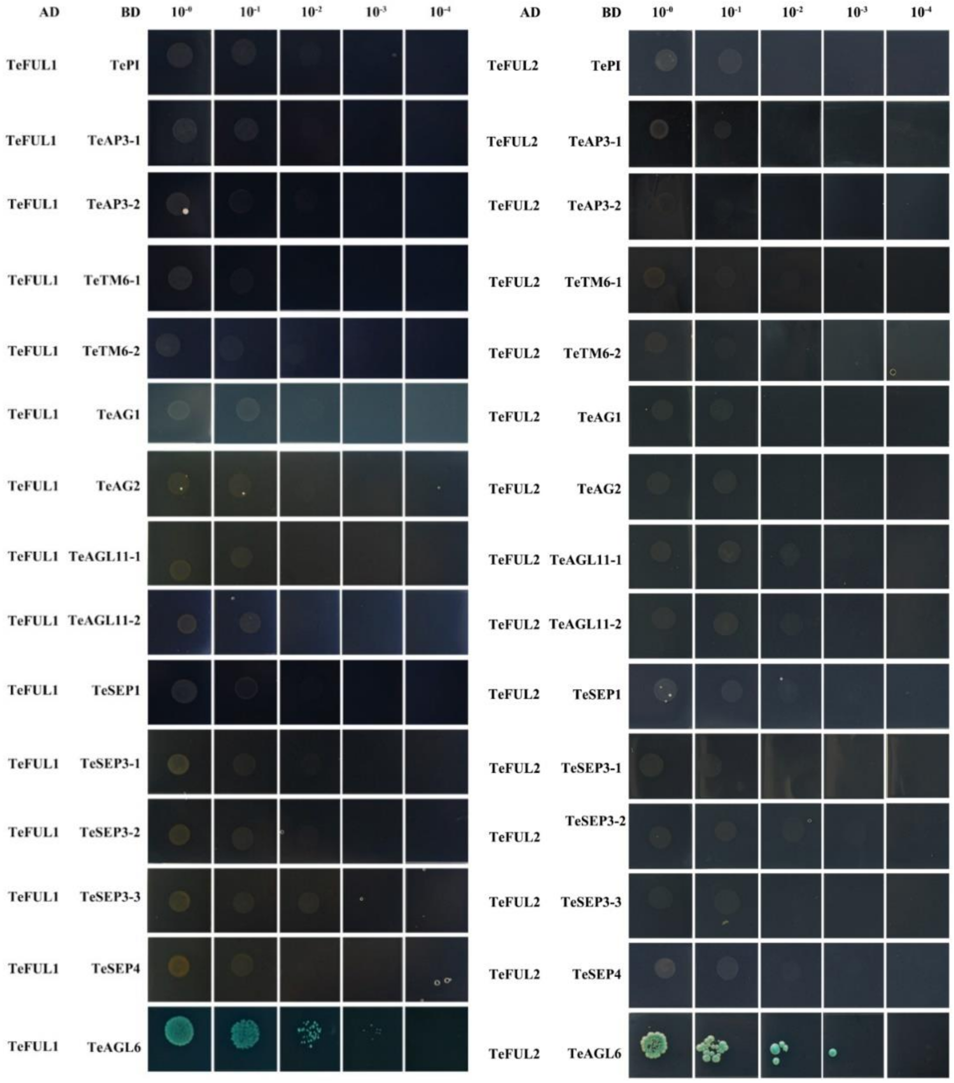

AD
BD
10⁻⁰
10⁻¹
10⁻²
10⁻³
10⁻⁴
TeFUL1 TePI
TeFUL1 TeAP3-1
TeFUL1 TeAP3-2
TeFUL1 TeTM6-1
TeFUL1 TeTM6-2
TeFUL1 TeAG1
TeFUL1 TeAG2
TeFUL1 TeAGL11-1
TeFUL1 TeAGL11-2
TeFUL1 TeSEP1
TeFUL1 TeSEP3-1
TeFUL1 TeSEP3-2
TeFUL1 TeSEP3-3
TeFUL1 TeSEP4
TeFUL1 TeAGL6
TeFUL2 TePI
TeFUL2 TeAP3-1
TeFUL2 TeAP3-2
TeFUL2 TeTM6-1
TeFUL2 TeTM6-2
TeFUL2 TeAG1
TeFUL2 TeAG2
TeFUL2 TeAGL11-1
TeFUL2 TeAGL11-2
TeFUL2 TeSEP1
TeFUL2 TeSEP3-1
TeFUL2 TeSEP3-2
TeFUL2 TeSEP3-3
TeFUL2 TeSEP4
TeFUL2 TeAGL6

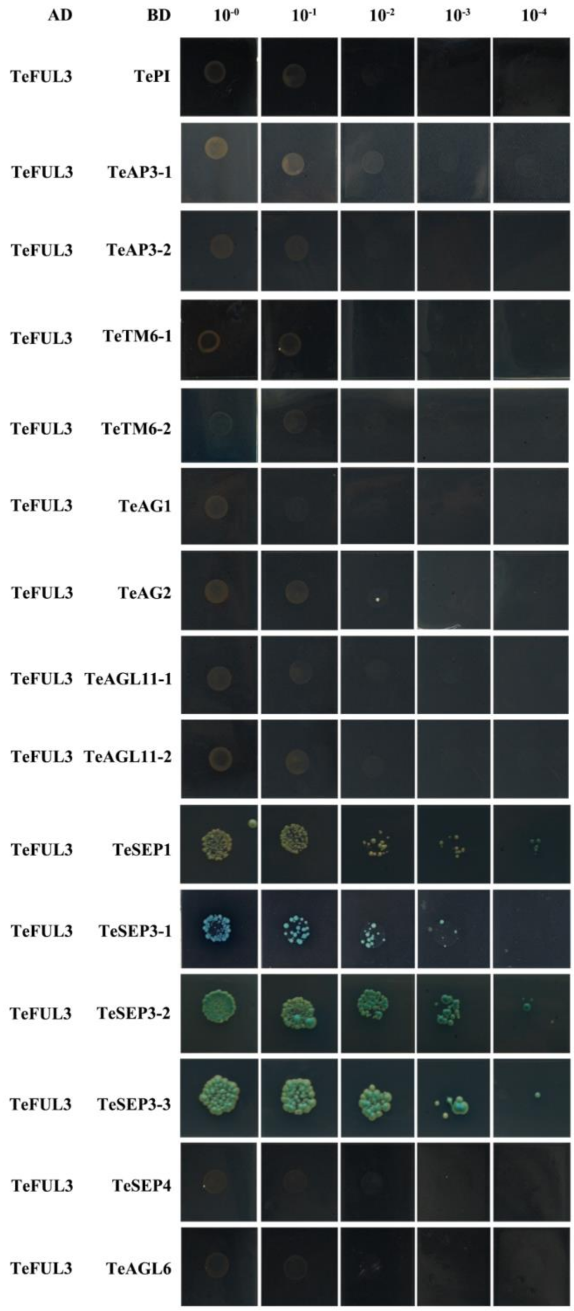
AD
BD
10^{-0}
10^{-1}
10^{-2}
10^{-3}
10^{-4}
TeFUL3 TePI
TeFUL3 TeAP3-1
TeFUL3 TeAP3-2
TeFUL3 TeTM6-1
TeFUL3 TeTM6-2
TeFUL3 TeAG1
TeFUL3 TeAG2
TeFUL3 TeAGL11-1
TeFUL3 TeAGL11-2
TeFUL3 TeSEP1
TeFUL3 TeSEP3-1
TeFUL3 TeSEP3-2
TeFUL3 TeSEP3-3
TeFUL3 TeSEP4
TeFUL3 TeAGL6

(c)

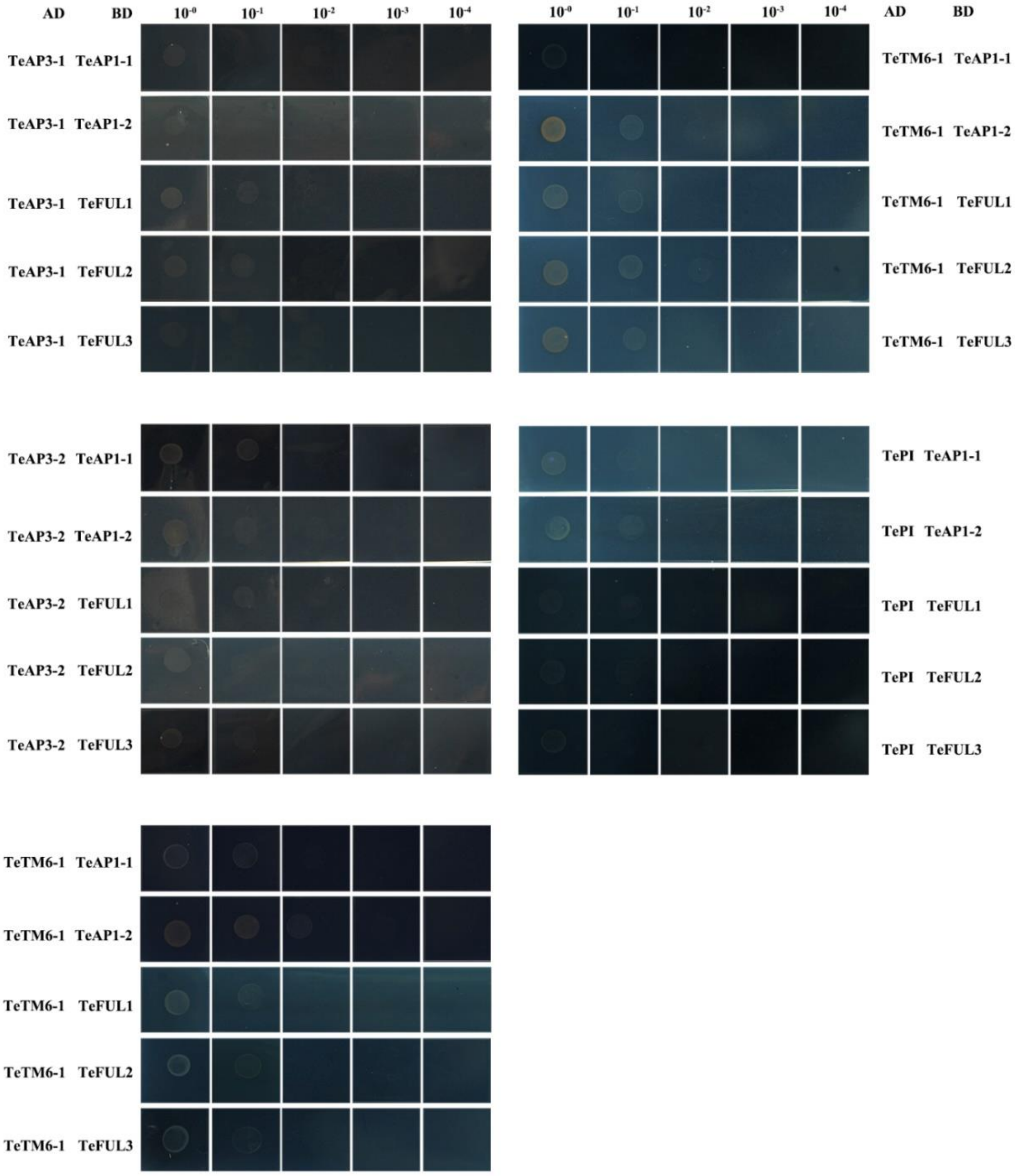
AD BD
10⁻⁰ 10⁻¹ 10⁻² 10⁻³ 10⁻⁴
TeAP3-1 TeAP1-1
TeAP3-1 TeAP1-2
TeAP3-1 TeFUL1
TeAP3-1 TeFUL2
TeAP3-1 TeFUL3
TeTM6-1 TeAP1-1
TeTM6-1 TeAP1-2
TeTM6-1 TeFUL1
TeTM6-1 TeFUL2
TeTM6-1 TeFUL3
TeAP3-2 TeAP1-1
TeAP3-2 TeAP1-2
TeAP3-2 TeFUL1
TeAP3-2 TeFUL2
TeAP3-2 TeFUL3
TePI TeAP1-1
TePI TeAP1-2
TePI TeFUL1
TePI TeFUL2
TePI TeFUL3

(d)

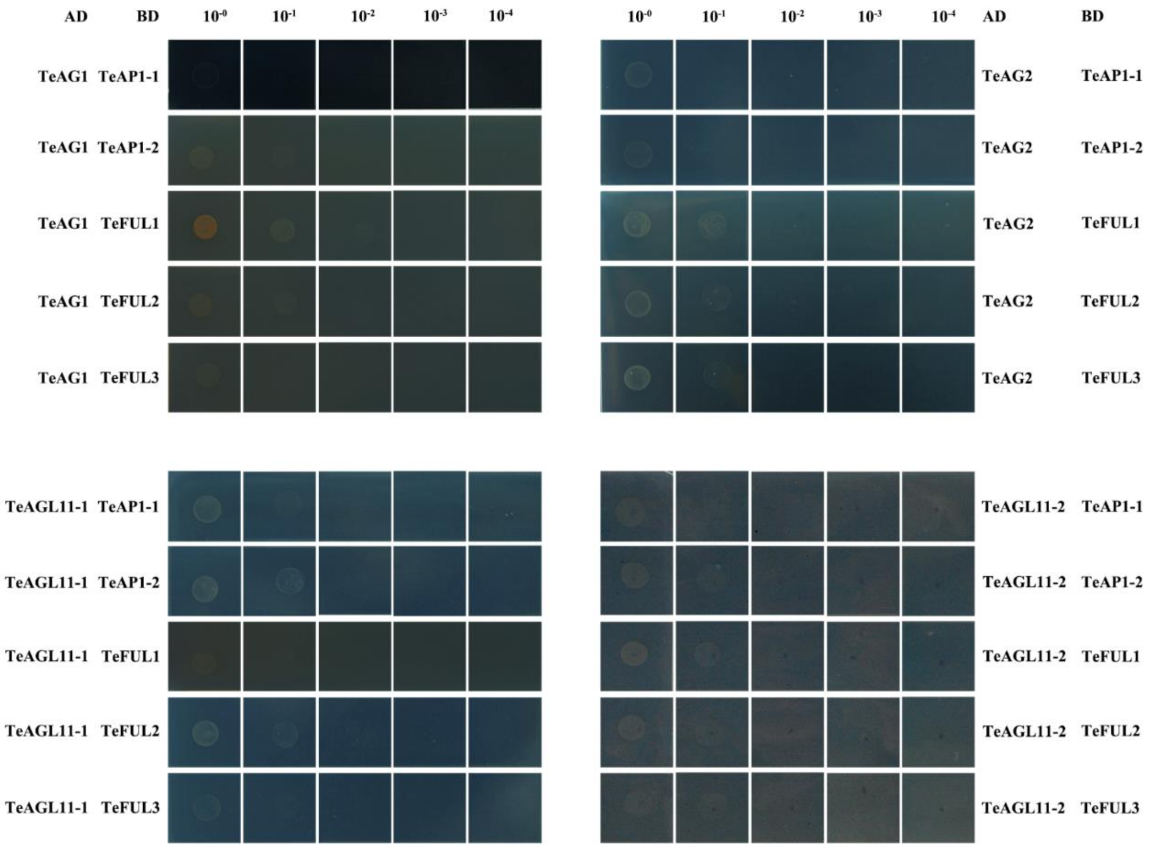

AD
BD
10^{-0}
10^{-1}
10^{-2}
10^{-3}
10^{-4}
TeAG1 TeAP1-1
TeAG1 TeAP1-2
TeAG1 TeFUL1
TeAG1 TeFUL2
TeAG1 TeFUL3
TeAG2 TeAP1-1
TeAG2 TeAP1-2
TeAG2 TeFUL1
TeAG2 TeFUL2
TeAG2 TeFUL3
TeAGL11-1 TeAP1-1
TeAGL11-1 TeAP1-2
TeAGL11-1 TeFUL1
TeAGL11-1 TeFUL2
TeAGL11-1 TeFUL3
TeAGL11-2 TeAP1-1
TeAGL11-2 TeAP1-2
TeAGL11-2 TeFUL1
TeAGL11-2 TeFUL2
TeAGL11-2 TeFUL3

(e)

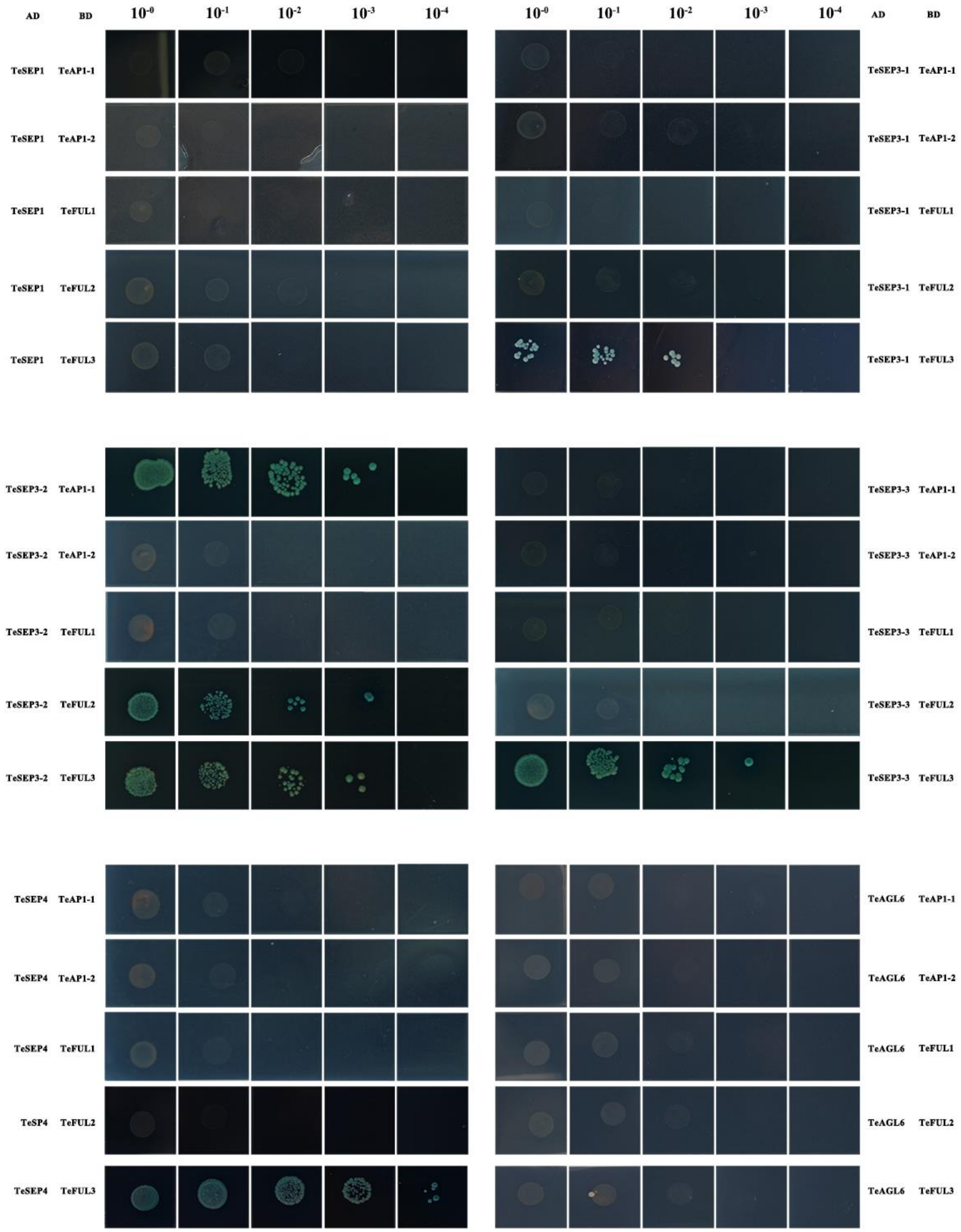

(f)

Figure S3. Interactions of TeAP1/FUL proteins with class A, B, C, D and, E proteins of marigold by yeast two-hybrid assays. (**a**) Assessing of Self-activation and auto-action of AD and BD constructs. **(b)** Interactions of TeAP1/FUL proteins with class B, C, D, and E proteins

of marigold by yeast two-hybrid assays. Ten-fold serial dilutions from 10-1 to 10-5 of each culture were spotted on the selected SD -Leu/-Trp/-His/-Ade plates with X-α-gal. **(c)** Interactions of TeAP1/FUL with class A, B, C, D, and E proteins of marigold by yeast two-hybrid assays. Ten-fold serial dilutions from 10^{-1} to 10^{-5} of each culture were spotted on the selected SD -Leu/-Trp/-His/-Ade plates with X-α-gal. Class B proteins: TeAP3-1, TeAP3-2, TePI, TeTM6-1, TeTM6-2. Class C proteins: TeAG1 and TeAG2 proteins; Class D proteins: TeAGL11-1 and TeAGL11-2; Class E proteins: TeSEP1, TeSEP3-1, TeSEP3-2, TeSEP3-3, TeSEP4 and TeAGL6 proteins. **(d)** Interactions of class B proteins and TeAP1/FUL proteins of marigold by yeast two-hybrid assays. Ten-fold serial dilutions from 10^{-1} to 10^{-5} of each culture were spotted on the selected SD -Leu/-Trp/-His/-Ade plates with X-α-gal. Class B proteins: TeAP3-1, TeAP3-2, TePI, TeTM6-1, TeTM6-2. **(e)** Interactions of class C and D proteins with TeAP1/FUL proteins of marigold by yeast two-hybrid assays. Ten-fold serial dilutions from 10^{-1} to 10^{-5} of each culture were spotted on the selected SD -Leu/-Trp/-His/-Ade plates with X-α-gal. Class C proteins: TeAG1 and TeAG2 proteins; Class D proteins: TeAGL11-1 and TeAGL11-2. **(f)** Interactions of class E proteins and TeAP1/FUL proteins of marigold by yeast two-hybrid assays. Ten-fold serial dilutions from 10^{-1} to 10^{-5} of each culture were spotted on the selected SD -Leu/-Trp/-His/-Ade plates with X-α-gal. Class D proteins: Class E proteins: TeSEP1, TeSEP3-1, TeSEP3-2, TeSEP3-3, TeSEP4 and TeAGL6 proteins.

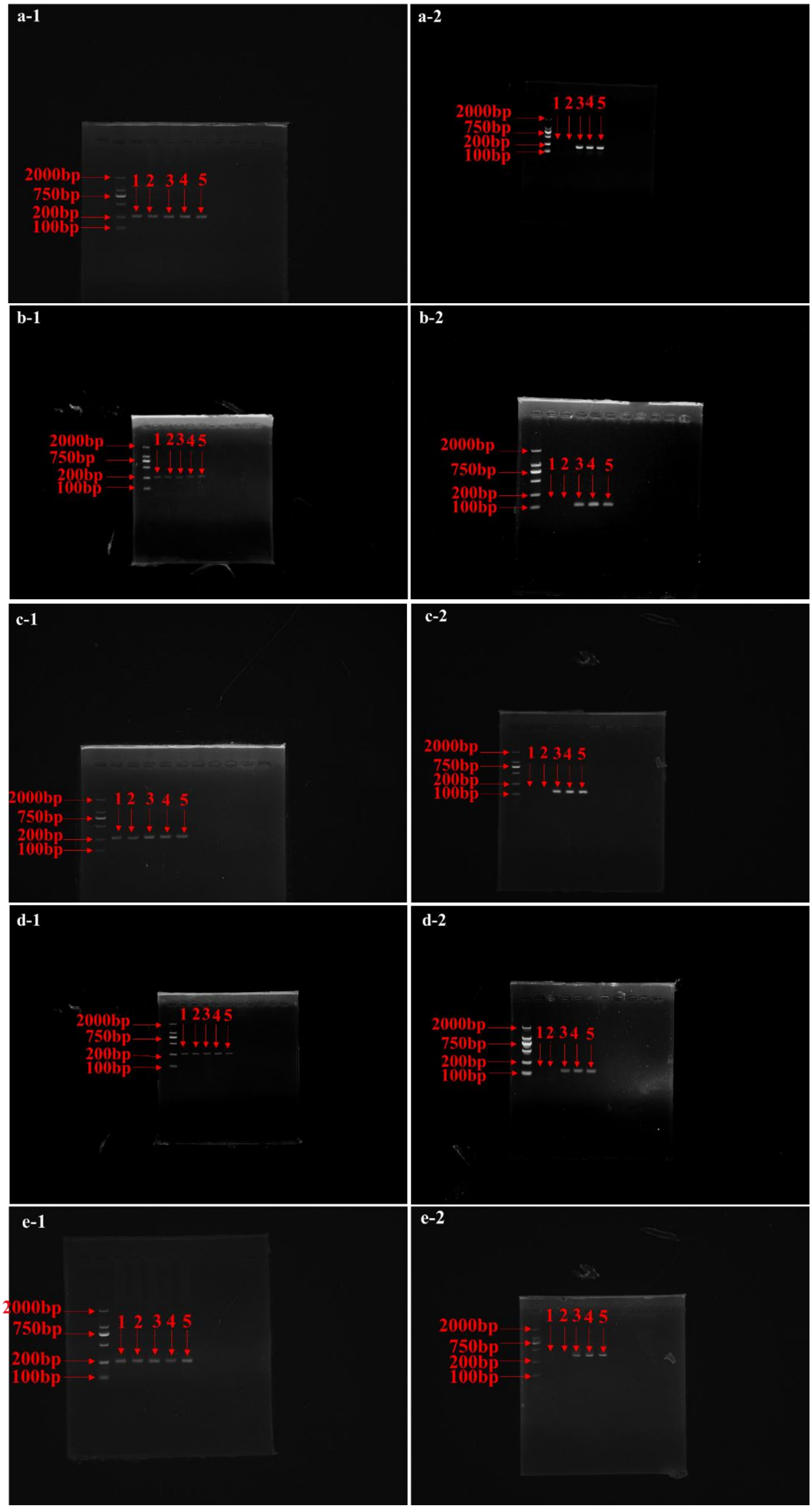
a-1
2000bp
750bp
200bp
100bp
1 2 3 4 5
a-2
2000bp
750bp
200bp
100bp
1 2 3 4 5
b-1
2000bp
750bp
200bp
100bp
1 2 3 4 5
b-2
2000bp
750bp
200bp
100bp
1 2 3 4 5
c-1
2000bp
750bp
200bp
100bp
1 2 3 4 5
c-2
2000bp
750bp
200bp
100bp
1 2 3 4 5
d-1
2000bp
750bp
200bp
100bp
1 2 3 4 5
d-2
2000bp
750bp
200bp
100bp
1 2 3 4 5
e-1
2000bp
750bp
200bp
100bp
1 2 3 4 5
e-2
2000bp
750bp
200bp
100bp
1 2 3 4 5

f

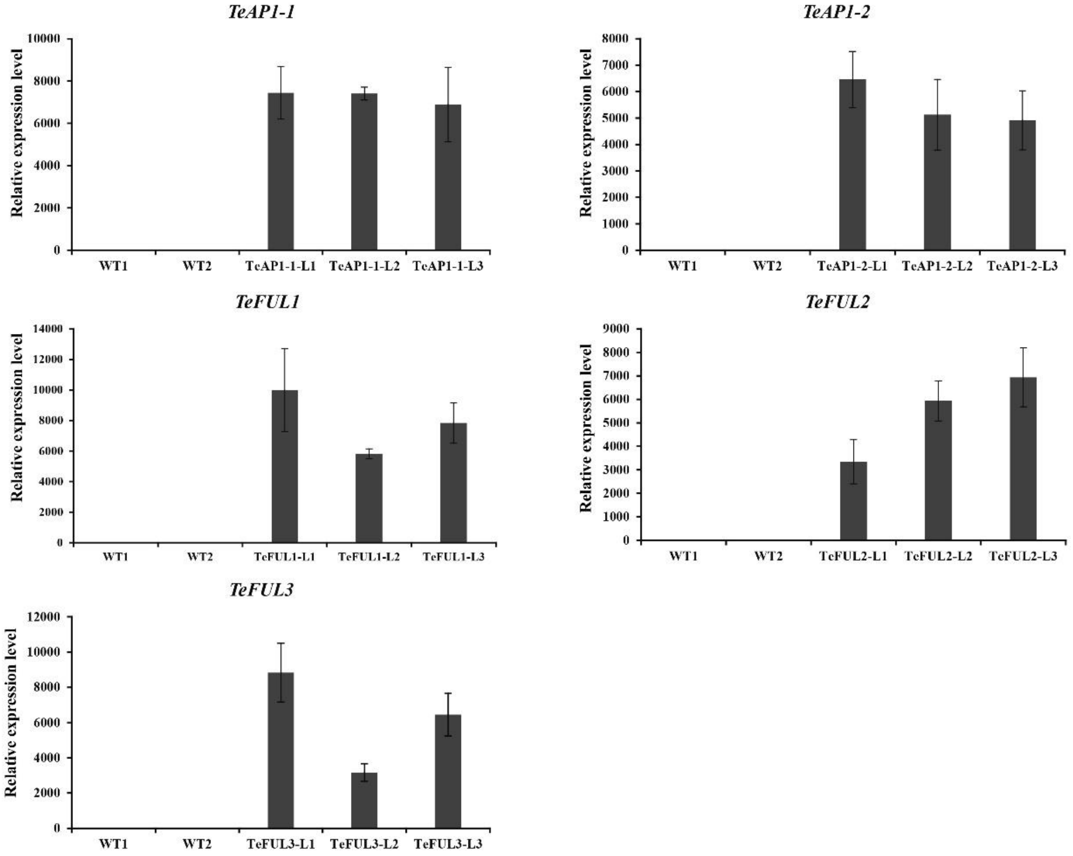

Figure S4. Expression of *TeAP1-1*, *TeAP1-2*, *TeFUL1*, *TeFUL2* and *TeFUL3* in seedlings of T_1 transgenic plants. (**a-e**) Expression of *TeAP1-1*, *TeFUL1*, and *TeFUL3* in seedlings of T1 transgenic lines by semi-quantitative RT-PCR. (**a**) Semi-qRT-PCR analysis of transgenes in wild-type Arabidopsis and *35S:TeAP1-1* transgenic plants. (**a-1**) The picture was the constitutive gene is Arabidopsis house-keeping gene *AtEF1α*. (**a-2**) The picture was the expression level of *TeAP1-1* in transgenic lines, the band size was 180 bp. The number of 1-5 indicate wild-type Arabidopsis plant 1, wild-type Arabidopsis plant 2, *35S:TeAP1-1* transgenic Arabidopsis line 1, *35S:TeAP1-1* transgenic Arabidopsis line 2, and *35S:TeAP1-1* transgenic Arabidopsis line 3, respectively. (**b**) Semi-qRT-PCR analysis of transgenes in wild-type Arabidopsis and *35S:TeAP1-2* transgenic plants. (**b-1**) The picture was the constitutive gene is Arabidopsis house-keeping gene *AtEF1α*. (**b-2**) The picture was the expression level of *TeTeAP1-2* in transgenic lines, the band size was 106 bp. The number of 1-5 indicate wild-type Arabidopsis plant 1, wild-type Arabidopsis plant 2, *35S:TeAP1-2* transgenic Arabidopsis line 1, *35S:TeAP1-2* transgenic Arabidopsis line 2, and *35S:TeAP1-1* transgenic Arabidopsis line 3, respectively. (**c**) Semi-qRT-PCR analysis of transgenes in wild-type Arabidopsis and *35S:TeFUL1* transgenic plants. (**c-1**) The picture was the constitutive gene is Arabidopsis house-keeping gene *AtEF1α*. (**c-2**) The picture was the expression level of *TeFUL1* in transgenic lines, the band size was 139 bp. The number of 1-5 indicate wild-type Arabidopsis plant 1, wild-type Arabidopsis plant 2, *35S:TeFUL1* transgenic Arabidopsis line 1, *35S:TeAFUL1* transgenic Arabidopsis line 2, and *35S:TeFUL1* transgenic Arabidopsis line 3, respectively. (**d**) Semi-qRT-PCR analysis of transgenes in wild-type Arabidopsis and *35S:FUL2* transgenic plants. (**d-1**) The picture was the constitutive gene is Arabidopsis house-keeping gene *AtEF1α*. (**d-2**) The

picture was the expression level of *TeFUL2* in transgenic lines, the band size was 127 bp. The number of 1-5 indicate wild-type Arabidopsis plant 1, wild-type Arabidopsis plant 2, *35S:TeFUL2* transgenic Arabidopsis line 1, *35S:TeFUL2* transgenic Arabidopsis line 2, and *35S:TeFUL2* transgenic Arabidopsis line 3, respectively. (**e**) Semi-qRT-PCR analysis of transgenes in wild-type Arabidopsis and *35S:FUL3* transgenic plants. (**e-1**) The picture was the constitutive gene is Arabidopsis house-keeping gene *AtEF1α*. (**e-2**) The picture was the expression level of *TeFUL3* in transgenic lines, the band size was 377 bp. The number of 1-5 indicate wild-type Arabidopsis plant 1, wild-type Arabidopsis plant 2, *35S:TeFUL3* transgenic Arabidopsis line 1, *35S:TeFUL3* transgenic Arabidopsis line 2, and *35S:TeFUL3* transgenic Arabidopsis line 3, respectively. (**f**) Expression of *TeAP1-1*, *TeAP1-2*, *TeFUL1*, *TeFUL2* and *TeFUL3* in seedlings of T_1 transgenic lines by quantitative real-time PCR.
